# Supplementary material for: WordCommentsAnalyzer: A windows software tool for qualitative research
Source: F1000Res. 2018 Sep 4;7:536. Originally published 2018 May 3. [Version 2] doi: 10.12688/f1000research.14819.2 (PMC6137411; doi:10.12688/f1000research.14819.2)
Supplement: Supplementary file 3 [file f1000research-7-17614-s0002.tgz › b2a79496-4ada-47c4-9682-a99ce867836d.zip!/Eight Iranian Health Journals/Yafteh _12.docx]

© Yafteh  . This work is licensed under CC BY 4.0 (https://creativecommons.org/licenses/by/4.0/).

Volume & Issue: دوره 17 - شماره 2

Title: بررسی موارد تأیید و عدم تأیید تقاضای سقط جنین درمانی در مراجعین پزشکی قانونی استان لرستان در سال 1392

http://yafte.lums.ac.ir/article-1-1966-fa.html

بحث و نتیجه‌گیری: سقط جنین به معنی سقط درمانی است و با افزایش موارد مجاز انجام سقط جنین درمانی، از انجام سقط‌های غیر قانونی کاسته خواهد شد و سبب افزایش سلامت زنان باردار می گردد. با افزایش میزان آگاهی کادر پزشکی از موارد مجاز سقط درمانی و قوانین مربوطه و راهنمایی صحیحتر زنان باردار، می‌توان به سلامت جامعه آنها کمک شایانی نمود. و همچنین، از مشکلات متخصصین زنان و زایمان نیز در این زمینه کاسته می شود. مواد و روش‌ها: در این مطالعه بصورت اپیدمیولوژیک، توصیفی- تحلیلی و مقطعی، کل درخواست های سقط جنین استان لرستان که در سال 1392 جهت دریافت مجوز به پزشکی قانونی شهرستان خرم آباد ارجاع شده بودند مورد بررسی قرار گرفتند، اطلاعات مربوط به این افراد در پرسشنامه ثبت شد و با استفاده از نرم افزار spss تجزیه و تحلیل گردید. یافته‌ها: از میان 205 مراجعه کننده در طول یک سال به 144 مورد مجوز سقط داده شده که از این میان، 88% به علت ناهنجاری جنینی و 12% به علت بیماری های مادر بود. فراوان ترین بیماری در جنین ها، اختلالات مغز و جمجمه و در مادران بیماری های قلبی عروقی و اختلالات هماتولوژیک بود. در این موارد، بیشترین فراوانی مربوط به گروه سنی 34-25 سال بود. از بین 61 مورد درخواست که موارد مجوز صادر نشده است سن بالای حاملگی با 26 مورد، شایع ترین علت رد درخواست سقط جنین و در بین این افراد نیز، بیماریهای مغز و جمجمه، شایع ترین علت درخواست سقط بود. مقدمه: با صدور مجوز سقط جنین در برخی موارد بیماریهای جنین و مادر که با تصویب قانون در سال 84 عملی شد، تحولی اساسی در سقط جنین درمانی در نظام سلامت ایران صورت گرفت. هر چند ممکن است مواردی از سقط غیرقانونی وجود داشته باشد ولی مطالعه ما بررسی سقط های قانونی استان لرستان، طی سال 1392 می باشد که منجر به افزایش آگاهی کادر پزشکی و درمانی در مورد اندیکاسیون های سقط درمانی، زمان انجام آن و پاسخ به سئوالات مربوطه است.

Title: مقایسه و ارتباط حمایت اجتماعی و استرس با رضایت از زندگی در سالمندان شهر خرم آباد در سال 1392

http://yafte.lums.ac.ir/article-1-1967-fa.html

مقدمه: سالمندی یک مسئله جهانی است که در آینده ای نزدیک به عنوان یکی از مهم ترین چالش های اجتماعی و رفاهی کشورهای در حال توسعه مطرح خواهد گردید. در این پژوهش حمایت اجتماعی و رضایت از زندگی در سالمندان ساکن در خانه سالمندان و همراه خانواده مقایسه شد. بحث و نتیجه‌گیری: بر اساس یافته های پژوهش حاضر، افزایش سطح حمایت های اجتماعی به ویژه حمایت خانوداگی و مقابله با عوامل استرس زا، باعث افزایش سطح رضایت مندی سالمندان از زندگی می شود. یافته‌ها: نتایج این پژوهش، حاکی از وجود رابطه معناداری (با ضریب همبستگی746/0)، بین حمایت اجتماعی و رضایت از زندگی در سالمندان بود. استرس نیز رابطه معنادار و معکوسی (با ضریب همبستگی383/0-) با رضایت از زندگی در سالمندان داشت (01/0< P). نتایج همچنین نشان داد که بین شاخص های رضایت از زندگی، استرس و حمایت اجتماعی در میان سالمندان با خانواده و سالمندان ساکن در خانه سالمندان تفاوت معناداری وجود دارد(01/0< P).  مواد و روش‌ها: این مطالعه اپیدمیولوژیک - تحلیلی بر روی نمونه ای 120 نفری از سالمندان زن و مرد ساکن شهر خرم آباد شامل 60 نفر سالمند با خانواده و 60 نفر سالمند دور از خانواده (زندگی در خانه سالمندان) که به روش تصادفی نظامدار و خوشه ای انتخاب شده بودند، انجام شد. برای سنجش رضایت از زندگی سالمندان، از پرسشنامه رضایت از زندگی نوگارتن و هوایرست (با پایایی 89/0) و برای سنجش حمایت و استرس از مقیاس حمایت و استرس اجتماعی دوک (با پایایی78/0) استفاده شد. داده های حاصل با استفاده از همبستگی پیرسون و یو من ویتنی مورد تجزیه و تحلیل قرار گرفتند. تمامی تحلیل ها در نرم افزار SPSS انجام شد.

Title: بررسی شیوع عفونت سل نهفته در کارکنان بیمارستان شهدای عشایر خرم آباد، 1394

http://yafte.lums.ac.ir/article-1-1968-fa.html

مقدمه: با وجود برنامه های گسترده کنترل جهانی بیماری سل، شیوع این بیماری در کشورهای توسعه نیافته و در حال توسعه همچنان بالاست. همچنین تعیین شیوع عفونت سل نهفته در کارکنان بیمارستان ها از اهمیت ویژه ای برخوردار است. بحث و نتیجه‌گیری: از بین 223 نفر شرکت کننده 40 نفر (18%) مبتلا به سل نهفته بودند که با توجه به تعداد بالای بیماران اسمیر مثبت بستری در این بیمارستان نشان دهنده نیاز جدی به اقدامات و برنامه های آموزشی فراگیرتر در جهت پیشگیری از ابتلا به این عفونت می باشد. یافته‌ها: تعداد 160 زن (7/71%)، 63 مرد (3/28%) در این مطالعه شرکت کردند که در این میان 172 نفر (1/77%) اندازه اندوراسیون کمتر از 5 میلی متر، 11 نفر (9/4%) اندازه اندوراسیون 5 تا کمتر از 10 میلی متر، 20 نفر (9%) اندوراسیون 10 تا 15 میلی متر و 20 نفر (9%) نیز اندوراسیون بالای 15 میلی متر داشتند. در این مطالعه بین جنس، سن، سابقه تماس با بیمار سل، سابقه کار و سل نهفته رابطه معناداری یافت نشد اما بین بخش فعالیتی، شغل کارکنان و سل نهفته رابطه معناداری وجود داشت. مواد و روش‌ها: طی یک مطالعه تحلیلی-توصیفی که در سال 1394 در بیمارستان عشایر خرم آباد بر روی کارکنان انجام شد، پس از اخذ شرح حال و کسب رضایت از کارکنان، تست پوستی توبرکولین (TST) انجام گرفته و نتایج آن 72-48 ساعت بعد قرائت و ثبت گردید. جهت بررسی اثر بوستر در افراد با نتایج TSTمنفی، تست یک هفته بعد تکرار گردید. معیار تشخیص سل نهفته تست توبرکولین با اندوراسیون بیشتر مساوی 10 میلی متر بود.

Title: بررسی میزان اثر بخشی آموزش مولفه های احیای قلبی ریوی بر ارتقاء آگاهی و مهارت های بالینی کارورزان بیهوشی در دانشگاه علوم پزشکی لرستان

http://yafte.lums.ac.ir/article-1-1970-fa.html

مقدمه: از آن جایی که تنها احیاء قلبی ریوی صحیح و مؤثر می تواند احتمال بقاء بیماران دچار ایست قلبی ـ تنفسی را بالا ببرد این مطالعه برای ارزیابی تأثیر آموزش مولفه های احیای قلبی ریوی در بخش بیهوشی بر ارتقاء آگاهی و مهارت های بالینی کارورزان بیهوشی در دانشگاه علوم پزشکی لرستان طراحی شد. بحث و نتیجه‌گیری: آموزش احیای قلبی ریوی طی دوره کارورزی بیهوشی منجر به افزایش واضح میزان آگاهی و مهارت کارورزان پزشکی می‌گردد. اضافه کردن این دوره به برنامه آموزشی کارورزان پزشکی در همه دانشگاه‌های علوم پزشکی ضروری به نظر می رسد. یافته‌ها: بعد از اتمام دوره کارورزی بیهوشی میانگین نمره آگاهی و مهارت کارورزان به طور واضحی، به ترتیب از 68/5 به 94/7 و از 65/10 به 45/23 افزایش یافت (0001/0= P). همچنین رابطه مستقیمی بین میزان آگاهی قبل از دوره بیهوشی با نمره آزمون پیش کارورزی دیده شد (0001/0= P) ولی ارتباط واضحی بین میزان مهارت بالینی با نمره آزمون پیش کارورزی مشاهده نشد. مواد و روش‌ها: در این مطالعه 10 ماهه 31 کارورز پزشکی دانشگاه علوم پزشکی لرستان مورد بررسی قرار گرفتند و همه کارورزان طی دوره 15 روزه کارورزی بیهوشی تحت آموزش تئوری احیای قلبی ریوی شامل BLS و ACLS و مهارت های بالینی مربوطه قرار گرفتند. میزان آگاهی و مهارت کارورزان قبل و بعد از دوره به وسیله پرسشنامه و ارزیابی عملی توسط دانشیار بخش بیهوشی ارزیابی گردید. این پرسشنامه شامل چهار قسمت مجزا 1- اطلاعات شخصی 2- سوالات تئوری جهت بررسی میزان دانش و آگاهی 3- بررسی مهارتهای عملی کارورز از دیدگاه خودش در دو زمان قبل و بعد از آموزش 4- بررسی مهارتهای عملی کارورز از دیدگاه دانشیار رشته بیهوشی در دو زمان قبل و بعد از آموزش احیای قلبی – ریوی بود.

Title: اثر عصاره برگ زیتون بر غلظت هورمونهای کورتیکوسترون و دئیدرواپی آندروسترون سرمی بعد از ایجاد هیپوپرفیوژن مغزی در رت های نر

http://yafte.lums.ac.ir/article-1-1971-fa.html

بحث و نتیجه‌گیری: در این مطالعه عصاره برگ زیتون با دوز mg/kg 300 موفق گردیده که از کاهش معنی دارغلظت کورتیکوسترون در نتیجه هیپوپرفیوژن مغزی جلوگیری کند و این نشان دهنده اثر حفاظتی عصاره برگ زیتون بر محور هیپوتالاموس- هیپوفیز – آدرنال می باشد و از آنجایی که عصاره برگ زیتون دارای اثرات مفید با طیف وسیع می باشد برای یافتن مکانیسم دقیق آن پیشنهاد می‌گردد در تحقیقات آینده عناصر موثره عصاره برگ زیتون بکار برده شود. مواد و روش‌ها: در این تحقیق از 35 راس رت ویستار نر استفاده گردید که به طور تصادفی به 5 گروه کنترل، هیپوپرفیوژن و گروههای هیپوپرفیوژن گاواژ شده با عصاره برگ زیتون بترتیب با دوزهای 100، 200 و 300 میلی گرم بر کیلوگرم وزن بدن حیوان تقسیم شدند. در گروه هیپوپرفیوژن حیوانات فقط تحت عمل جراحی بستن دائم دو رگ کاروتید به فاصله یک هفته قرار گرفتند و در گروه کنترل حیوانات تحت عمل جراحی کاذب، باز شدن غلاف کاروتید بدون بستن رگ ها، قرار گرفتند. عمل گاواژ حیوانات بمدت 25 روز صورت گرفت. در انتها با ایجاد بیهوشی عمیق در حیوانات نمونه خونی آنها را گرفته و غلظت هورمونهای کورتیکوسترون و DHEA موجود در سرم توسط کیتهای الیزا اندازه گیری گردید. یافته‌ها: هیپوپرفیوژن مغزی سبب کاهش معنی داری در غلظت کورتیکوسترون سرم در مقایسه با گروه کنترل گردید (01/0< P)، اما تیمار با عصاره برگ زیتونmg/Kg 300 قادر بوده است که میزان کورتیکوسترون را به مقدار طبیعی ( گروه کنترل) برگرداند. تفاوت معنی داری در غلظت DHEA بین گروهها مشاهده نشد. مقدمه: هیپوپرفیوژن مزمن مغزی یک مورد پاتوفیزیولوژیک شایع می باشد که معمولاً در شرایطی نظیر دمانس رگی و آلزایمر اتفاق می افتد که هر دوی این بیماریها توسط اختلالات حافظه تشخیص داده می شوند. تشخیص مکانیسمهای مشخص که در سلسله وقایع موجود از هیپوپرفیوژن مزمن تا کاهش حافظه نقش دارد می توانند هدف بالقوه ای برای درمان های موثر باشد هدف از مطالعه حاضر با توجه به اثرات مفید آنتی اکسیدانی و ضد التهابی عصاره برگ زیتون، بررسی اثرات عصاره برگ زیتون بر غلظت هورمونهای کورتیکوسترون و دئیدرواپی آندروسترون (DHEA) سرمی بعد از ایجاد هیپوپرفیوژن مغزی در رت می باشد.

Title: شیوع نوروپاتی محیطی در بیماران مبتلا به دیابت مراجعه کننده به مرکز دیابت شهرخرم آباد در سال 1394

http://yafte.lums.ac.ir/article-1-1972-fa.html

مقدمه: نوروپاتی دیابتی مهمترین عارضه دیابت می باشد که باعث ناتوانی در بیماران می شود. شواهد حاکی از آن می باشد که شناسایی زودرس نوروپاتی می‌تواند باعث کاهش زخم پای دیابتی و قطع عضو شود. این مطالعه با هدف بررسی شیوع نوروپاتی محیطی در بیماران دیابتی طراحی گردید. بحث و نتیجه‌گیری: نتایج این مطالعه بر اهمیت غربالگری در شناسایی زود هنگام نوروپاتی در بیماران دیابتی تأکید دارد. پرسشنامه میشیگان با توجه به سادگی، قابلیت اجرا و کم هزینه بودن آن، برای شناسایی افراد در معرض خطر نوروپاتی مناسب به نظر می رسد. یافته‌ها: شیوع نوروپاتی دیابتی در بیماران بررسی شده 28 درصد بود. بین طول مدت بیماری و نوروپاتی ارتباط مستقیم مشاهده گردید که به لحاظ آماری معنادار بود (02/0= P) در حالی که بین نوروپاتی و سایر متغیرها ارتباط معناداری مشاهده نگردید. مواد و روش‌ها: مطالعه به صورت توصیفی مقطعی در سال 1394 طراحی شد و نمونه ها به صورت تصادفی از بیماران دیابتی مراجعه کننده به مرکز دیابت خرم آباد انتخاب شدند. ابزار جمع آوری داده ها شامل اطلاعات دموگرافیک، آنتروپومتریک و پرسشنامه میشیگان بود که از دو قسمت مصاحبه (علایم نوروپاتی) و معاینه تشکیل شده است. برای آنالیز داده ها از آمار توصیفی و تحلیلی استفاده گردید.

Title: تعیین اثر درمان و عوامل مؤثر بر بقا در بیماران مبتلا به سرطان معده تحت عمل جراحی و تحت شیمی‌درمانی

http://yafte.lums.ac.ir/article-1-1973-fa.html

بحث و نتیجه‌گیری: توجه به ویژگی‎های دموگرافیک و پاتولوژیک بیماران و تعیین ریسک فاکتورهای مرتبط در موفقیت درمان و افزایش بقا مؤثر است ولیکن برای تعیین اثر علی درمان بایستی از کارآزمایی‎های بالینی کنترل شده برای حذف اثرات مرتبط با درمان استفاده کرد. مواد و روش‌ها: یک مطالعه همگروهی تاریخی بر روی 181 بیمار تحت شیمی‎درمانی (گروه 1) و 201 بیمار تحت جراحی (گروه 2) انجام شد. اثر متغیرهای دموگرافیکی، کلینیکی و پاتولوژیکی بر بقا در دو گروه درمانی با استفاده از آزمون لگ-رتبه و مدل مخاطرات متناسب کاکس ارزیابی شد. تجزیه و تحلیل با استفاده از نرم‌افزارSPSS16 انجام شد. یافته‌ها: 56 بیمار گروه اول و 69 بیمار گروه دوم تا پایان مطالعه فوت شدند. میانه‌ی بقای بیماران گروه اول و دوم بترتیب 19 و 28 ماه بود. تأًثیر متغیرهای سن تشخیص و درجه تمایزیافتگی تومور بر بقای گروه اول و جنس و مرحله بیماری بر بقای گروه دوم معنی‌دار شدند (05/0P<). مقدمه: جراحی به عنوان مهمترین روش درمان بیماران سرطان معده مطرح بوده و شیمی‎درمانی و رادیوتراپی نیز به عنوان درمان کمکی پس از جراحی در این بیماران حین عمل و پس از عمل برای کاهش عود موضعی و متاستاز مورد استفاده قرار می‎گیرد. هدف این مطالعه مقایسه بقا و ویژگی‎های بیماران جراحی شده با بیمارانی که شیمی‎درمانی دریافت کرده‎اند می‎باشد.

Title: کاربرد مدل فرا نظری در رفتار پیشگیری از پوکی استخوان (فعالیت فیزیکی) در زنان ۳۰-۵۰ سال خرمآباد: یک مطالعه مبتنی بر مدل فرا نظریه ایی

http://yafte.lums.ac.ir/article-1-1974-fa.html

مقدمه: پوکی استخوان یک اولویت مهم بهداشتی در ایران و جهان است. اخیراً پیشگیری از پوکی استخوان، هدف بسیاری از متخصصان امر سلامت شده است. تغییر رفتار یکی از استراتژیهای قدرتمند جهت پیشگیری از پوکی استخوان است. مطالعه حاضر با هدف تعیین کاربرد مدل فرا نظری در رفتار پیشگیری از پوکی استخوان (فعالیت فیزیکی) در زنان ۳۰-۵۰ سال خرمآباد طراحی شده است بحث و نتیجه‌گیری: نتایج مطالعه نشان داد که از میان سازههای مورد بررسی مدل فرا نظری، خودکارآمدی تنها سازه پیشگویی کننده اتخاذ رفتار پیشگیری از پوکی استخوان هست. بنابراین یافتههای این پژوهش میتواند بهعنوان پایهای برای مداخلات آموزشی در زمینه تغییر رفتار جهت پیشگیری از پوکی استخوان، مورد استفاده متولیان امر سلامت قرار گیرد. یافته‌ها: میانگین، انحراف معیار، سنی افراد مورد مطالعه ۳/۷±۷۲/۳۸ سال و میانگین و انحراف معیار فعالیت فیزیکی سبک هفتگی برحسب دقیقه برابر با ۴۰۰/۵۶±۸۳/۳۸ بود. همچنین نتایج نشان داد که سازه خودکارآمدی بالاترین توان پیشگوییکنندگی رفتار پیشگیرانه را دارد. مواد و روش‌ها: در این مطالعه توصیفی تحلیلی مقطعی، ۲۶۹ زن سنین 5۰-30 سال بر اساس معیارهای پژوهش بهصورت تصادفی خوشهای و سیستماتیک از بین کلیه مراکز بهداشتی درمانی خرمآباد انتخاب شدند. ابزار گردآوری اطلاعات پرسشنامههای روا و پایای اطلاعات فردی، مراحل تغییر رفتار، تعادل تصمیمی و خودکارآمدی فعالیت فیزیکی بود. تجزیه و تحلیل اطلاعات با استفاده از آزمون کای دو، ضریب همبستگی اسپیرمن و رگرسیون لجستیک انجام شد.

Title: تأثیر تزریق درون صفاقی کریستال ‌مت بر روی محور هیپوفیز – گناد در موش‌های صحرایی نر بالغ

http://yafte.lums.ac.ir/article-1-1975-fa.html

مقدمه: سوء استفاده از آمفتامین‌های روان گردان (کریستال مت و اکستازی) در سالهای اخیر در بین نوجوانان و جوانان ایرانی رواج یافته است. این مواد اثرات مخرب بسیاری را در اندام‌های بدن بجا می‌گذارند. از این رو این مطالعه به منظور تعیین اثر کریستال‌ مت روی محور هورمونی هیپوفیز - گناد موش صحرایی نر بالغ انجام شد. بحث و نتیجه‌گیری: با توجه به نتایج بدست آمده از سنجش‌های هورمونی می‌توان نتیجه گرفت که کریستال مت دارای اثرات مخرب بر روی محور هورمونی هیپوفیز – گنادی جنس نر دارد. یافته‌ها: غلظت سرمی هورمون تستوسترون در گروه‌های تجربی دریافت کننده دوز 10 و 15 میلی گرم بر کیلوگرم وزن بدن نسبت به گروه کنترل، افزایش معنی داری نشان داد (05/0P<). غلظت سرمی هورمون‌های LH و FSH در گروه ‌های تجربی دریافت کننده دوز 5، 10 و 15 کاهش معنی‌داری را نشان داد (05/0P<). مواد و روش‌ها: 28 سر موش صحرایی نر نژاد ویستار به چهار گروه کنترل و گروه‌های دریافت کننده کریستال مت با دوز 5، 10 و 15 میلی-گرم بر کیلوگرم وزن بدن تقسیم شدند. گروه‌های تجربی به مدت 7 روز، کریستال‌ مت را به شیوه درون صفاقی دریافت نمودند و پس از 7 روز، خون از بطن چپ قلب موش‌ها گرفته شده و پارامترهای مورد نظر (تستوسترون، LH و FSH) توسط کیت الایزا اندازه گیری شد. آنالیز داده-های حاصل، با استفاده از نرم‌افزار آماری 16- SPSS صورت گرفت. مقایسه گروه‌های آزمایشی مختلف توسط آزمون آماری تحلیل واریانس یک طرفه (ANOVA) انجام شد و 05/0P< معنی‌دار در نظر گرفته شد.

Title: اثربخشی مداخله دلبستگی محور با روش پسخوراند ویدیویی بر کاهش بازنمایی های منفی و اضطراب جدایی کودکان

http://yafte.lums.ac.ir/article-1-1976-fa.html

مقدمه: با توجه به اهمیت دلبستگی در پدیدآیی اضطراب جدایی، هدف این مطالعه بررسی اثربخشی مداخله دلبستگی محور با روش پسخوراند ویدیویی بر کاهش بازنمایی های منفی و مشکلات اضطراب جدایی در کودکان پیش دبستانی بود. بحث و نتیجه‌گیری: با توجه به یافته ها به نظر می رسد مداخله دلبستگی محور موجب افزایش حساسیت مادرانه و این تغییر همراه با کاهش بازنمایی های منفی کودک به واسطه تعاملات ترمیم کننده، توانسته اضطراب جدایی کودکان را کاهش دهد. یافته‌ها: یافته ها نشان دهنده اثر بخشی مداخله دلبستگی محور بر افزایش حساسیت مادرانه، کاهش بازنمایی های منفی کودک و همچنین علائم اضطراب جدایی در کودکان گروه آزمایش بود. میانگین نمرات گروه آزمایش در مقایسه با گواه در تمام متغیرها به طور معناداری کاهش یافته و این کاهش در پیگیری باقی مانده بود. مواد و روش‌ها: طرح پژوهش نیمه آزمایشی با پیش آزمون، پس آزمون و پیگیری بود. برای این منظور، 21 زوج مادر و کودک (11 زوج در گروه آزمایش و 10 زوج در گروه گواه) از دو مرکز تخصصی مشاوره کودک بر مبنای معیارهای ورود مطالعه انتخاب شدند. گروه آزمایش بین 8 تا 10 جلسه مداخله انفرادی دلبستگی محور و گروه گواه مداخله روابط محور دیگری را به منظور مقایسه اثربخشی دریافت کردند. شرکت کنندگان در سه مرحله پیش آزمون، پس آزمون و پیگیری (3 ماه بعد) با استفاده از ابزارهای درجه بندی رفتار مادرانه Q-SORT، مجموعه داستانهای مک آرتور و پرسشنامه علائم مرضی کودکان ارزیابی شدند.

Title: بکارگیری روشی نوین در درمان آلزایمر القاء شده توسط استرپتوزوتوسین در رت های نر نژاد ویستار همراه با کاهش عوارض هماتوکریت

http://yafte.lums.ac.ir/article-1-1977-fa.html

بحث و نتیجه‌گیری: این نتایج پیشنهاد داد که تزریق STZ در بطن های جانبی مغز موجب آسیب شدید در حافظه و یادگیری مشابه بیماری آلزایمر می شود ولی اریتروپویتین از این آسیب همراه با کاهش در هماتوکریت جلوگیری می کند. مواد و روش‌ها: جهت ایجاد مدل آلزایمری در رت ها، استرپتوزوتوسین در بطن های طرفی مغزی تزریق گردید. 2 هفته پس از القاء آلزایمر داروی اریتروپویتین با دوز های متفاوت به صورت سیسترنامگنایی جهت درمان آلزایمر همراه با کاهش عوارض جانبی این دارو تزریق گردید. بعد از دوره درمان همه رت ها تحت مطالعه رفتاری آزمون یادگیری اجتنابی غیر فعال توسط دستگاه شاتل باکس و سنجش هماتوکریت خون قرار گرفتند. یافته‌ها: هرچند اریتروپویتین تأثیر معنی داری بر بهبود فرآیند های یادگیری و حافظه در هر دو گروه کنترل و شم نداشت ولی باعث ایجاد تفاوت معنی داری در بهبود این بیماری نسبت به گروه STZ(استرپتوزوتوسین) شد که این درمان با کاهش هماتوکریت نسبت به گروه دوز U/Kg۵۰۰۰ همراه شد. مقدمه: بیماری آلزایمر یک بیماری شایع نورودژنراتیو پیش رونده ای است که منجر به زوال عقل و نابودی سلول های مغزی به ویژه در ناحیه هیپوکامپ می شود. به تازگی مشخص شده اریتروپویتین با عمل حفاظت عصبی خود باعث بهبود در عملکردهای شناختی در طول این بیماری می گردد. هدف این مطالعه یافتن روشی جایگزین نسبت به روش مرسوم تزریق صفاقی برای تحویل اریتروپویتین به مغز توسط دور زدن سد خونی مغزی است.

Title: شیوع سرولوژیک توکسوپلاسموز در اهدا کنندگان خون مرکز انتقال خون همدان در سال 1392

http://yafte.lums.ac.ir/article-1-1978-fa.html

بحث و نتیجه‌گیری: چون غربالگری خون از نظر توکسوپلاسما در سازمان انتقال خون انجام نمی گیرد، لذا بررسی اهدا کنندگان در سازمان انتقال خون برای جلوگیری از خطرات احتمالی می تواند مورد توجه کارشناسی قرار گیرد. مواد و روش‌ها: در این مطالعه توصیفی- مقطعی سرم 540 نفر از اهدا کنندگان خون در سازمان انتقال خون همدان به طور تصادفی انتخاب و با روش ELISA عیار آنتی بادیهای IgM و IgG تعیین و فراوانی برخی فاکتورهای خطر آلودگی به انگل با پرسشنامه ای گردآوری و آنالیز شد. یافته‌ها: طبق نتایج بدست آمده در آزمایش الایزا از مجموع 540 نفر که 518 نفر آنها مرد و 19 نفر زن (و 3 نفر ذکر نشده) بودند، 294 نفر ) 4/54% ( دارای آنتی بادی IgG و 10 نفر (9/1% ) دارای آنتی بادی IgM بر علیه توکسوپلاسما گوندی بودند. مقدمه: ﺗﻮﻛﺴﻮﭘﻼﺳﻤﻮز ﺑا ﺷﻴﻮع ﺟﻬﺎﻧﻲ، ایجاد سقط و ﻋﻮارض شدید در ﻧﻮزادان ﻣﺘﻮﻟـﺪ ﺷـﺪه از ﻣﺎدرانی ﻛﻪ در دوران ﺑﺎرداری ﺑـﻪ ﺗﻮﻛﺴﻮﭘﻼﺳـﻤﻮز حاد ﻣﺒـﺘﻼ می شوند، ﺑـﻪ ﻋﻨـﻮان ﻳﻜـﻲ از ﺷﺎیع ترﻳﻦ ﻋﻔﻮﻧﺘﻬﺎی ﻓﺮﺻﺖ ﻃﻠﺐ در ﺑﻴﻤﺎران دﭼﺎر ﻧﻘﺺ اﻳﻤﻨﻲ اﻫﻤﻴﺖ وﻳﮋه ای دارد. گرچه راه اصلی انتقال تماس با گربه و خوردن موادگوشتی می باشد، اما چون در مرحله حاد عفونت انگل در تمام مایعات بدن وجود دارد، اگر در این مرحله خون اهدا شود انتقال انگل محتمل است. هدف این مطالعه بررسی شیوع آنتی بادیهای ضد توکسوپلاسما گوندی در اهدا کنندگان خون سازمان انتقال خون همدان بوده است.
